# Supplementary figures and images for: Evaluation of the diagnostic value of the Modified Evan’s Blue Dye Test for assessing aspiration in tracheostomized critically ill patients: A systematic review and meta-analysis
Source: PLoS One. 2026 May 13;21(5):e0349092. doi: 10.1371/journal.pone.0349092 (PMC13170877; doi:10.1371/journal.pone.0349092)

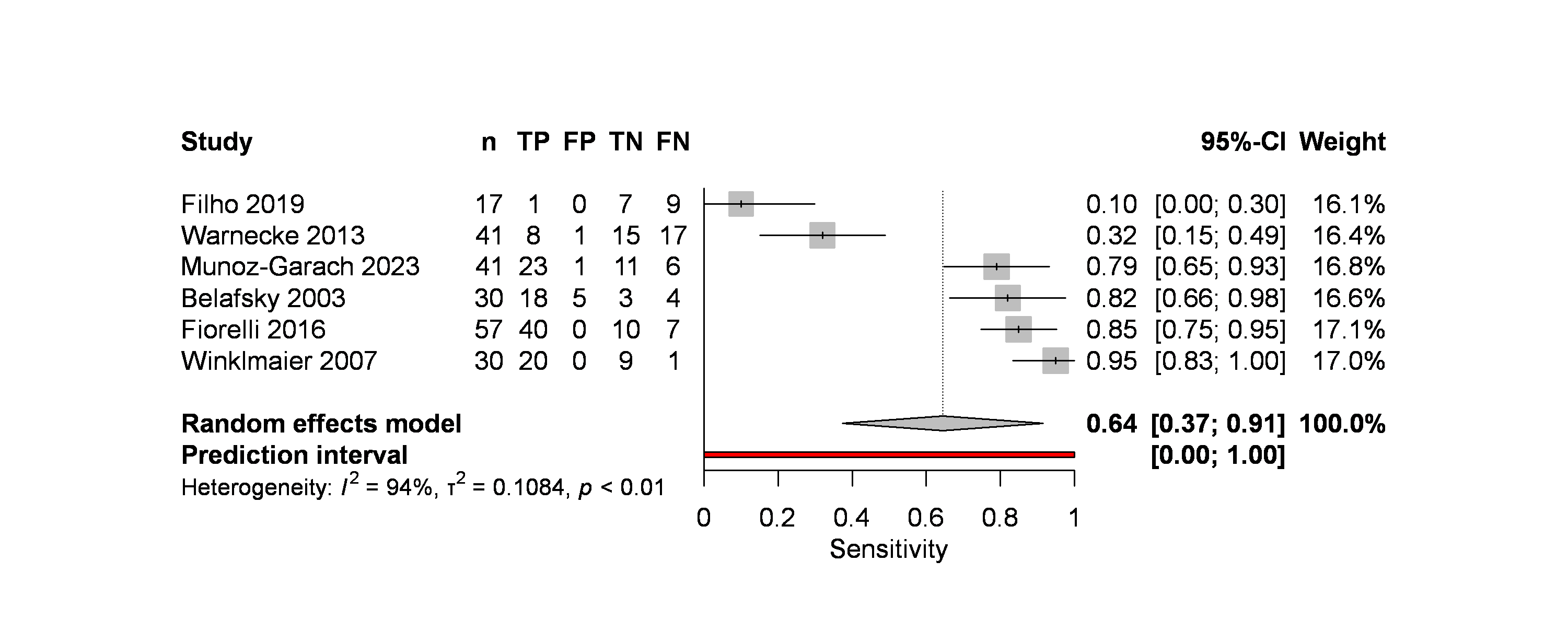

Supplement: S1 Fig — (TIF) [file pone.0349092.s001.tif]

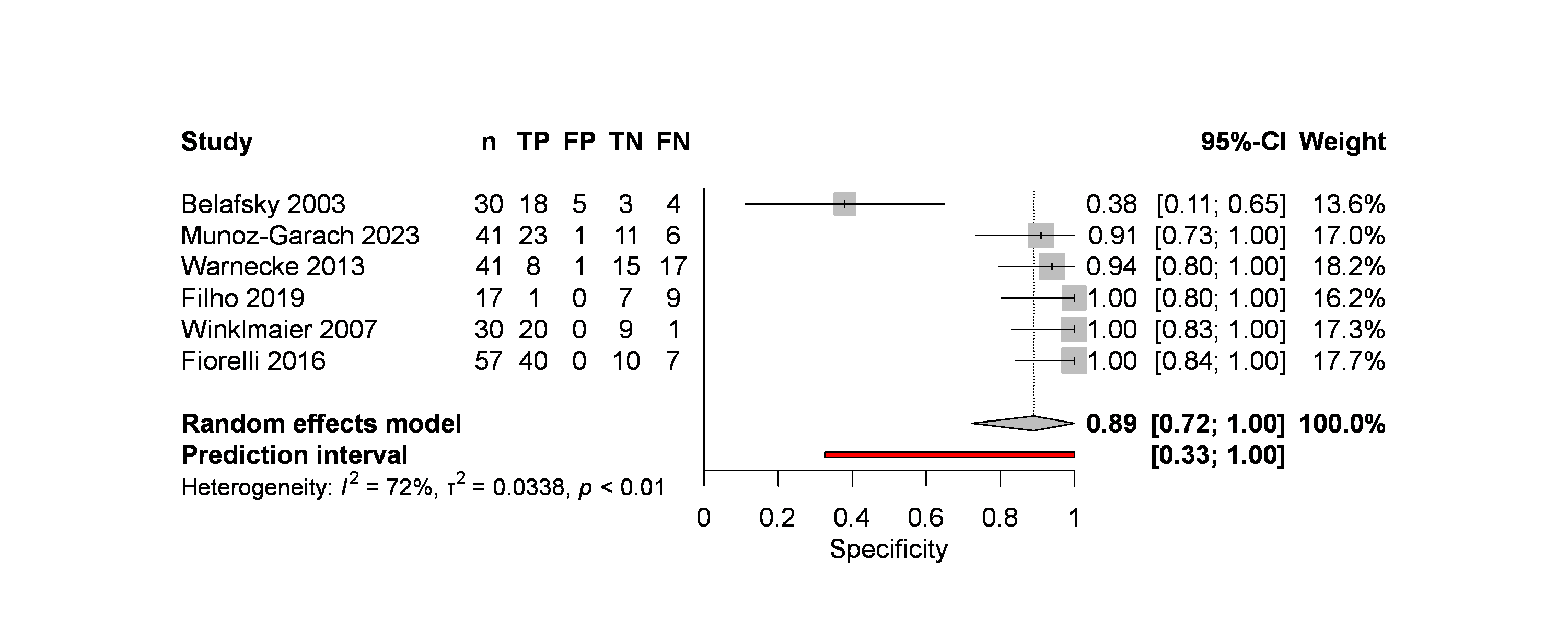

Supplement: S2 Fig — (TIF) [file pone.0349092.s002.tif]

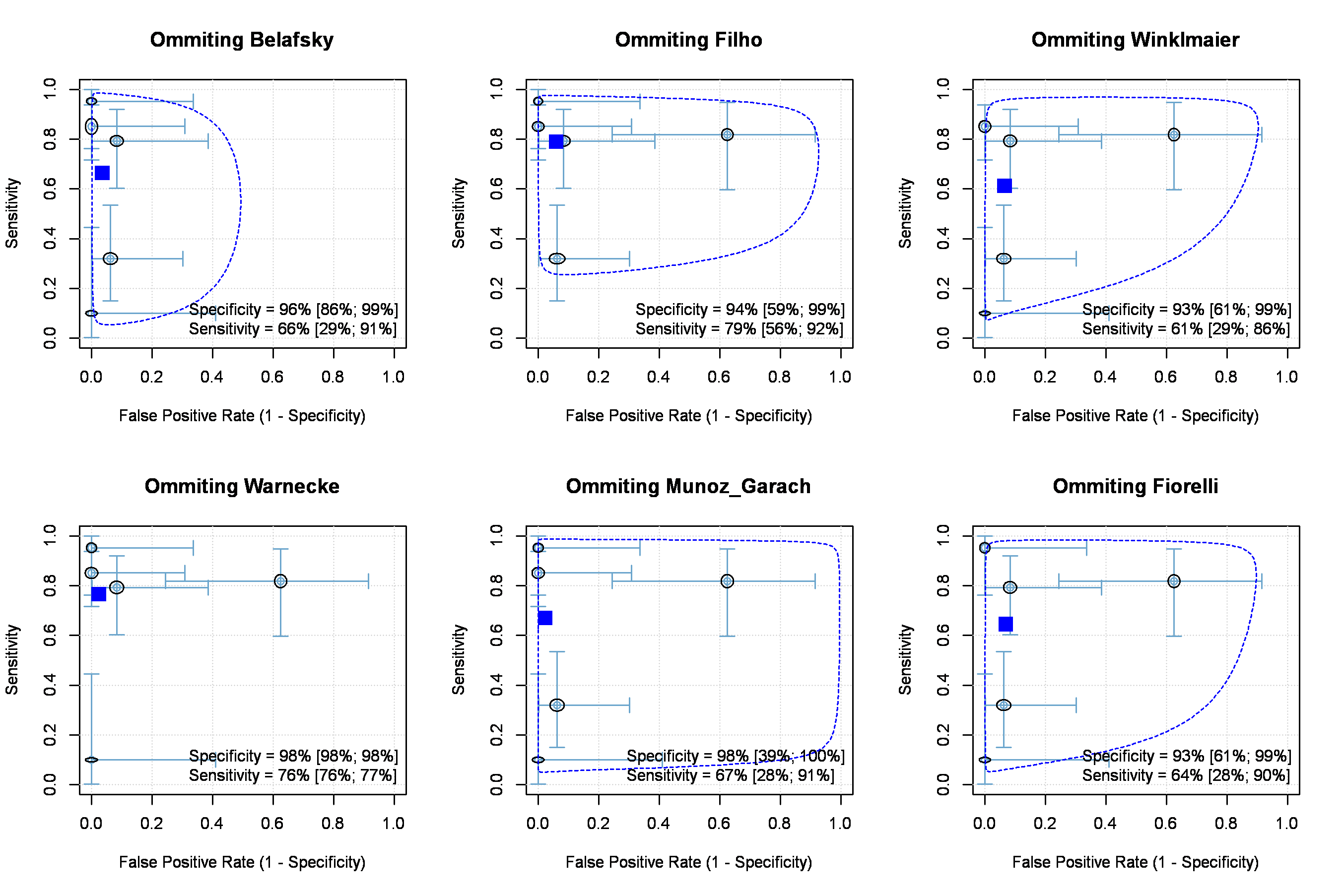

Supplement: S3 Fig — (TIF) [file pone.0349092.s003.tif]

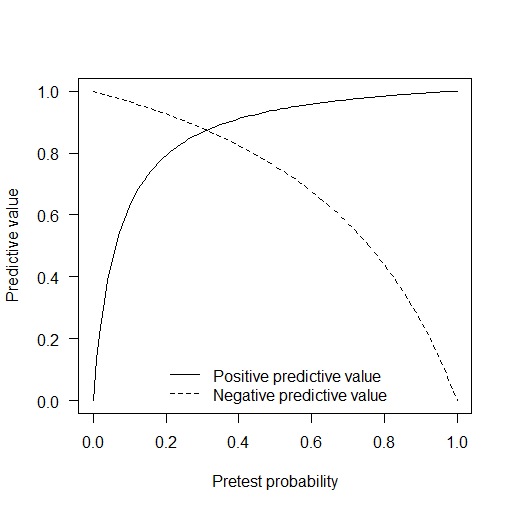

Supplement: S4 Fig — (TIF) [file pone.0349092.s004.tif]
