# Supplementary material for: Evaluation of the diagnostic value of the Modified Evan’s Blue Dye Test for assessing aspiration in tracheostomized critically ill patients: A systematic review and meta-analysis
Source: PLoS One. 2026 May 13;21(5):e0349092. doi: 10.1371/journal.pone.0349092 (PMC13170877; doi:10.1371/journal.pone.0349092)
Supplement: S1 Table — (DOCX) [file pone.0349092.s005.docx]

| **Study(first author, publication year)** | **Cuff deflated** | **Ventillated (n)** | **Ventilatory mode** | **Consistencies trialed** | **Time interval between MEBDT and FEES** |
| --- | --- | --- | --- | --- | --- |
| Belafsky et al., 2003[8] | yes | yes/no (10/20) | "minimized" pressure support | 3x15 ml ice chips swallowing, performed 3 times on 3 separate occasions separated by at least 1 hour | within 24 hours |
| Filho et al., 2019 [36] | yes | no | not applicable | 20 ml honey-like consistency | within one session |
| Winklmaier et al., 2007[37] | cuffed and uncuffed tubes (cuffed was deflated) | no | not applicable | 1^st^: 5 ml colored, artificial saliva 2^nd^: 1x 5 ml colored, artificial saliva 3^rd^: 1x15 ml colored, artificial saliva 4^th^ to 6^th^: colored water: 2x5 ml (swallow 4 and 5), and 1x15 ml (swallow 6) colored water. Suction was performed after the swallowing trials with saliva, after the swallowing examination with water, and 5 min after the examination was finished | FEES directly following MEBDT |
| Warnecke et al., 2013[38] | no information | weaned completely from mechanical ventilation | not applicable | teaspoon of jelly then a teaspoon of water | within one session |
| Munoz-Garach et al., 2023[42] | yes | weaned completely from mechanical ventilation | not applicable | 5 ml of water | FEES 6 and 24 hours after the MEBDT |
| Fiorelli et al., 2016 [24] | yes | weaned completely from mechanical ventilation | not applicable | water (amount not specified) | FEES was performed at least 24 hours following the last MEBDT |

**S1 Table:** **Methodological assessment of studies included.**
