# Supplementary material for: Evaluation of the diagnostic value of the Modified Evan’s Blue Dye Test for assessing aspiration in tracheostomized critically ill patients: A systematic review and meta-analysis
Source: PLoS One. 2026 May 13;21(5):e0349092. doi: 10.1371/journal.pone.0349092 (PMC13170877; doi:10.1371/journal.pone.0349092)
Supplement: Attachment — Submitted filename: Response to reviewers.doc [file pone.0349092.s010.doc]

**Response to Reviewers**

We appreciate the reviewers for their careful reading of our manuscript and for their valuable and constructive suggestions, which have helped us improve the clarity and accuracy of our work. All major revisions have been highlighted in grey in the revised manuscript (“Revised Manuscript With Changes Highlighted”). Deleted text has been retained using strikethrough where appropriate.

**Reviewer 1**

**Comment**

**Reviewer’s comment:**

*“In the abstract, the authors claim that "[they] conducted a systematic review and meta-analysis to investigate the effectiveness of MEBDT in detecting aspiration in tracheostomized critically ill patients." However, they say that the, "aim of this study was to evaluate the diagnostic accuracy of MEBDT in comparison with FEES in the assessment of aspiration in critically ill patients with tracheostomy." While similar, these two aims are different. They should either be addended to be the same, or a primary and secondary aim should have been set a priori to the study.”*

**Response:**

We thank the reviewer for this important and constructive comment. We agree that the original wording suggested two slightly different study aims. To address this issue, we revised the manuscript to consistently define a single and clear a priority objective. Specifically, we aligned the abstract with the stated aim of the study and clarified that the primary objective of this systematic review and meta-analysis was to evaluate the diagnostic accuracy of the Modified Evan’s Blue Dye Test (MEBDT) in comparison with Fiberoptic Endoscopic Evaluation of Swallowing (FEES) for the assessment of aspiration in tracheostomized critically ill patients. The term “effectiveness” was replaced to ensure conceptual clarity and to accurately reflect the diagnostic test accuracy framework of the study.

**Changes made:**

- The aim-related sentence in the abstract was revised by replacing “effectiveness” with terminology reflecting diagnostic accuracy compared with FEES.
- The aim of the study is now stated consistently across the abstract and the main manuscript (page 4, lines 53-57).

**Comment 1**

**Reviewer’s comment:**

*“1. page 13, line 76: "MEBDT should be performed in all critically ill tracheostomized patients." However, authors state that, "It is important to emphasize that PPV and NPV, and the conclusions based on them, are only valid for populations where the prevalence is similar to those in the studies examined (between 60-80%, which already represent quite heterogeneous prevalences). Therefore, conclusions based on MS do not apply to patient populations where the prevalence of aspiration is, for example, 10% (where PPV would be around 50% and NPV around 90%) or to a population with 90% prevalence (PPV ~100%, NPV ~20%)," on page 23. This is a major caveat, and a major flaw of their listed key point.”*

**Response:**

We thank the reviewer for this important and well-founded comment. We agree that the original wording of the key point may have been overly broad and did not sufficiently reflect the important limitations related to disease prevalence and the interpretation of predictive values. To address this concern, we revised the relevant section to clarify the prevalence-dependent nature of PPV and NPV and to avoid overly generalized recommendations regarding the use of MEBDT. The revised text now explicitly emphasizes that predictive values should be interpreted within the context of prevalence ranges comparable to those observed in the included studies and that conclusions cannot be directly extrapolated to populations with substantially lower or higher aspiration prevalence. This modification ensures consistency between the key points and the methodological limitations discussed later in the manuscript.

**Changes made:**

 The statement suggesting universal application of MEBDT was revised to avoid overgeneralization and to reflect the prevalence-dependent interpretation of predictive values.

 The section discussing PPV and NPV was expanded and clarified to explicitly highlight the dependence of predictive values on disease prevalence and the limited generalizability of these results to populations with substantially different prevalence rates (page16, lines 255-269 & page page 22, lines 389-398).

 The key message was revised to ensure full consistency with the methodological considerations discussed in the Discussion section.

**Comment 2**

**Reviewer’s comment:**

*„2. page 26, lines 303-308: "This suggests the application of different consistencies (water, saliva, or honey-like), different (5-10-20 ml) and repeated quantities of material to be swallowed. In this case, a PPV of 100% indicates that, in these studies, all patients who tested positive for aspiration using MEBDT were confirmed to have aspiration, meaning that the test identifies aspirates of such consistencies with high accuracy." This is not what it means. Accuracy can not be explained by one parameter like PPV. For instance, this test has a high PPV but a moderate sensitivity, indicating that it's not perfect at detecting all positive cases. This is an important fact and it is not consistent with a "high accuracy" at all.”*

**Response:**
We thank the reviewer for this important and technically well-founded comment. We fully agree that overall diagnostic accuracy cannot be inferred from a single parameter such as positive predictive value alone, and that high PPV does not imply high sensitivity or comprehensive case detection. To address this issue, we revised the manuscript to avoid the term “high accuracy” when referring solely to PPV. The revised text now clearly distinguishes between overall diagnostic accuracy and the reliability of positive test results. Specifically, we clarify that a PPV of 100% indicates that all positive MEBDT findings in these studies represented true aspiration events across the tested bolus consistencies, reflecting high reliability for aspiration confirmation rather than overall diagnostic accuracy.

**Changes made:**

- The wording implying “high accuracy” based solely on PPV was removed.
- The text was revised to explicitly state that PPV reflects the reliability of positive test results and does not capture overall diagnostic accuracy or sensitivity (page 20-21, lines 343-346).
- The interpretation of MEBDT performance was revised to align with standard diagnostic test accuracy principles, particularly by avoiding the interpretation of PPV as a measure of overall diagnostic accuracy.

**Comment 3**

**Reviewer’s comment:**

*„3. page 23, line 242: "However, for NPV and PPV, heterogeneity values were 0% with p = 0.5 and p = 0.95, respectively (Figs. 3 and 4). However, due to the low number of articles, these metrics do not provide much relevant information." I don't know what to say about this because at least half of the author's argument throughout the paper is that the positive predictive value being near 100% demonstrates high accuracy and/or is the basis for their argument in favor of widespread MEBDT use as a screening tool. What is the purpose of writing a paragraph about a heterogeneity analysis that "does not provide much relevant information."*

**Response:**
We thank the reviewer for this thoughtful and well-reasoned comment. We agree that presenting a heterogeneity analysis while simultaneously stating that it does not provide relevant information may be confusing and does not add meaningful interpretative value, especially in the context of a limited number of included studies. To address this concern, we removed this paragraph from the manuscript. This revision avoids potential inconsistency and improves the clarity and coherence of the results section by focusing on analyses that meaningfully contribute to the interpretation of MEBDT performance.

**Changes made:**

The paragraph describing heterogeneity analyses of PPV and NPV with limited interpretative value was removed from the Results section (page 17, lines 278-280).

**Comment 4**

**Reviewer’s comment:**

*„4. The authors reference the use of local anesthetic during FEES. While it is true that local anesthetic may be used during the procedure, it is not necessary and should be counted against its use in clinical practice. Some literature and practice experts suggests that anesthesia worsens the diagnostic ability of FEES, and recommend against its use.”*

**Response:**
We thank the reviewer for the comment regarding the use of local anesthesia during FEES. In accordance with the literature and clinical guidelines, we have revised the text to clarify that anesthesia is generally avoided during the procedure, as it may compromise the sensory aspects of swallowing and increase the risk of aspiration.

**Changes made:**

The sentence describing the use of local anesthesia during FEES was revised (page 19, lines 308-315) to clarify that anesthesia is generally avoided, as it may impair sensory assessment of swallowing and increase the risk of aspiration.

**Comment**

**Reviewer’s comment:**

„There are numerous places in the manuscript that are clearly missing information/words or used inconsistent formatting. Some examples included but are not limited to

1.page 12, lines 61-62: "Six out of XXX screened studies were included," where the XXX was not updated.

2.page 16, lines 124-125: "Pediatric population, patients without tracheostomy tube, and patients with contraindications to MEBDT and FEES were excluded," where "population" and "tube" should be plural.

3.page 16, lines 129-130: "...on 24th November 2023, and repeated it with the same search key and strategy on June 25, 2025."

4.page 25, lines 279-280: "FEES is positive for aspiration if saliva, food, or any liquid trapped under the vocal cords [28]," where the word "is" is missing between liquid and trapped.”

**Response:**

We thank the reviewer for carefully identifying these issues. We have thoroughly revised the manuscript to correct missing information, grammatical inaccuracies, and formatting inconsistencies. All examples raised by the reviewer were addressed, and the relevant sections were edited accordingly to improve clarity and accuracy.

**Changes made:**

The total number of screened studies was updated to **2227** (“Six out of 2227 screened studies were included”) (page 4).

Pluralization and wording were corrected in the exclusion criteria to improve grammatical accuracy and clarity (page 8).

The description of the search strategy was revised to clearly state the initial search date and the subsequent repeated search using consistent terminology and formatting (page 8).

The missing verb in the definition of aspiration during FEES was added (“…any liquid **is** trapped under the vocal cords”) (page 19).

**Comment**

**Reviewer’s comment:**

**“**There are many other places in the manuscript that were understandable, but clearly not written to the standard of an accredited scientific journal publication. I realize the authors are not from a predominately English-speaking country, but these issues should be addressed.

1. page 14, lines 103-104: "As transfer of the patient is inevitable for VFSS, our decision was to investigate the other two methods, applicable at the bedside."

2. page 28, lines 353-355: As for limitations, only a small number of cases were registered, 216 in total. In addition, only six articles could be included, all of which were observational studies, and reported significantly heterogenous practices."”

**Response:**
We thank the reviewer for this constructive comment. We agree that improvements in language clarity and academic style were necessary. As we did not have access to a native English-speaking editor, the manuscript was carefully revised by the authors to enhance grammatical accuracy, sentence structure, and overall readability, with particular attention to the examples highlighted by the reviewer. The cited sentences were rewritten to meet the linguistic and stylistic standards expected of an accredited scientific journal.

**Changes made:**

 The sentence referring to patient transfer for VFSS was rewritten for improved clarity and academic tone (page 6, lines 106-108).

 The limitations section was revised and restructured to improve clarity, grammatical correctness, and precision while preserving the original content (page 23, lines 403-411).

**Reviewer 2**

**Comment**

**Reviewer’s comment:**

*“Methods: Looking at the 6 studies included in the review, there was only 2/6 of the studies that had a study period, which were Winklmaier et al and Fiorelli et al, others were either unstated or non-specific. A pooled negative predictive value of 59% is indicative of a variable sensitivity of the use MEBDT which is a major limitation. When a negative result, i.e no blue dye is found, rather than completely ruling out aspiration, it may be due to presence of small aspirations or silent aspirations, where there are no cough reflex present. As well as the high specificity reported, 95.42% (95% CI [67.38%, 99.53%]) merely shows the ability to detect patients who do not aspirate, rather than being as good a diagnostic tool as FEES. This is because FEES is standardized in its uses as opposed to MEBDT, which makes comparism and drawing conclusions about studies with a variety of protocols quite challenging.
The risk of bias and GRADE assessment was high for 4/6 papers, which raises issues and concerns about the applicability of the findings of these publications.”*

**Response:**

We thank the reviewer for this comprehensive and insightful comment. We agree with these concerns and have revised the manuscript to more clearly and explicitly acknowledge the methodological limitations and diagnostic constraints highlighted. Specifically, the Limitations section was expanded to address the small overall sample size, the observational nature of the included studies, the substantial heterogeneity in clinical practice, and the fact that a clearly defined study period was reported in only two of the six included studies, limiting methodological transparency and comparability. In addition, we emphasized the lack of a uniformly standardized MEBDT protocol in contrast to FEES, which may contribute to methodological variability across studies.

Furthermore, the Conclusion was revised to clearly state that, although the high specificity and positive predictive value of MEBDT support its reliability in confirming aspiration when positive, the moderate pooled sensitivity indicates that a negative MEBDT result cannot reliably exclude aspiration. Accordingly, FEES remains the reference standard, and further prospective validation and expert consensus are required to define the standardized use and appropriate clinical role of MEBDT within diagnostic pathways.

**Changes made:**

- The Limitations section was expanded to address the small sample size, observational study designs, heterogeneity of clinical practice, limited reporting of study periods, and the lack of standardized MEBDT protocols (page 23, lines 403-411).
- The Conclusion was revised to clarify the implications of moderate sensitivity and negative predictive value and to explicitly state that a negative MEBDT result does not reliably exclude aspiration, reinforcing the role of FEES as the reference standard (page 25-26, lines 428-437).
