# Supplementary material for: Evaluation of the diagnostic value of the Modified Evan’s Blue Dye Test for assessing aspiration in tracheostomized critically ill patients: A systematic review and meta-analysis
Source: PLoS One. 2026 May 13;21(5):e0349092. doi: 10.1371/journal.pone.0349092 (PMC13170877; doi:10.1371/journal.pone.0349092)
Supplement: Attachment — Submitted filename: Reviewer Attachments for Manuscript Number PONE-D-25-53361.docx [file pone.0349092.s009.docx]

The revised manuscript still requires substantial revision to ensure that the clinical claims are strictly aligned with the meta-analytic results. While the pooled positive predictive value (PPV) is high, the pooled sensitivity is only moderate, and the pooled negative predictive value (NPV) is low (at around 59%). This means that a negative MEBDT result cannot reliably rule out aspiration in critically ill patients with tracheostomies. Therefore, if the intention is to safely exclude the risk of aspiration, the manuscript should avoid describing MEBDT as a 'screening' test.

Additionally, the manuscript should explicitly emphasise that the PPV depends on the prevalence and that the very high PPV observed is likely influenced by the high prevalence of aspiration in the included cohorts. Finally, given the heterogeneity of protocols, the lack of standardisation, and the very low GRADE certainty, any proposed clinical decision algorithm should be removed, or, if retained, clearly presented as hypothetical rather than as practice guidance. Overall, the manuscript may only be suitable if it is reframed as an adjunctive rule-in bedside signal (when positive), with clear guidance that negative results warrant further instrumental assessment (e.g. FEES) in high-risk settings.
