# Supplementary material for: Evaluation of the diagnostic value of the Modified Evan’s Blue Dye Test for assessing aspiration in tracheostomized critically ill patients: A systematic review and meta-analysis
Source: PLoS One. 2026 May 13;21(5):e0349092. doi: 10.1371/journal.pone.0349092 (PMC13170877; doi:10.1371/journal.pone.0349092)
Supplement: Attachment — Submitted filename: Response to reviewers_090426.doc [file pone.0349092.s011.doc]

**Response to Reviewers**

We sincerely thank the reviewers for their careful reading of our manuscript and for providing valuable and constructive suggestions, which have greatly helped us improve the clarity and accuracy of our work. All major revisions are highlighted in yellow in the revised manuscript (“Revised Manuscript With Changes Highlighted”), and deleted text has been retained using strikethrough where appropriate.

**Clinical claims aligned with meta-analytic results**

**Reviewer comment:** The manuscript should avoid describing MEBDT as a screening test due to moderate sensitivity and low NPV.

**Response:**

We thank the reviewer for this important comment. We agree and have revised the manuscript to ensure that the clinical interpretation is fully aligned with the meta-analytic findings.

Specifically, MEBDT is now consistently described as an adjunctive rule-in bedside tool rather than a screening test. We explicitly state that negative results cannot reliably exclude aspiration and require further instrumental assessment (e.g., FEES) in high-risk patients.

These revisions have been implemented in the Abstract (final sentence), Introduction (clinical context paragraph), Discussion (first paragraph), Key Points, and Conclusion, and the term “screening test” has been removed or avoided throughout the manuscript where clinically inappropriate.

**Positive predictive value (PPV) and prevalence**

**Reviewer comment:** PPV depends on prevalence, and the observed high PPV may be influenced by high prevalence in included cohorts.

**Response:**

We thank the reviewer for highlighting this important methodological consideration. We agree and have clarified the dependence of predictive values on prevalence in both the Results and Discussion sections.

We now explicitly report the high prevalence of aspiration (approximately 58–82%) in the included studies in the Results (Negative and positive predictive values section, first paragraph).

We further clarify that the high pooled PPV (95%) is influenced by this prevalence in the Discussion (Effectiveness and Limitations of MEBDT section).

In addition, we have included a new figure (Figure 5) illustrating how PPV and NPV vary across a wide range of prevalence values (described in the Results, final paragraph of the predictive values section). We also emphasize that these predictive values are directly applicable only to populations with similar prevalence.

**Clinical decision algorithm**

**Reviewer comment:** Proposed algorithms should either be removed or clearly presented as hypothetical.

**Response:**

We thank the reviewer for this suggestion. In accordance with this recommendation, the clinical decision algorithm has been removed from the manuscript.

**Emphasis on heterogeneity and certainty**

**Reviewer comment:** Highlight protocol heterogeneity and low certainty.

**Response:**

We agree with the reviewer and have strengthened the manuscript accordingly.

We now more explicitly describe the substantial heterogeneity in MEBDT protocols - including differences in bolus consistency, quantity, timing relative to FEES, and patient ventilation status - in the Discussion (Methodology and Implication variability in MEBDT section).

Statistical heterogeneity is reported in the Results (Publication bias and heterogeneity section).

Furthermore, we emphasize that the overall GRADE certainty of evidence is low in the Results (Risk of bias and GRADE assessment section), and we reinforce these limitations in the Strengths and limitations section.

**Summary of clinical implications**

**Response:**

We have revised the Key Points and Conclusion to provide a clear and clinically consistent summary.

**In the Key Points, we state that:**

- MEBDT can serve as a rule-in bedside signal for aspiration when positive

- Negative results cannot exclude aspiration (low NPV)

- Further instrumental assessment is required after negative results

These points are further strengthened in the Discussion (Effectiveness and Limitations section).

In the Conclusion, we summarize that MEBDT should be considered an adjunctive bedside tool, that predictive values may be influenced by prevalence, and that negative results require confirmation with instrumental assessment.

We have also revised the title of the manuscript to better reflect the focus on diagnostic accuracy and the cautious clinical interpretation of findings.

**We sincerely thank the reviewers again for their thoughtful and constructive comments, which have helped us improve the clarity and accuracy of our manuscript.**

Best regards,

Eszter Szőke
